# Factors contributing to the ceiling effect of the EQ-5D-5L: an analysis of patients with prostate cancer judged “no-problems”

**DOI:** 10.1007/s11136-019-02316-4

**Published:** 2019-10-03

**Authors:** Hideki Murasawa, Takayuki Sugiyama, Yuki Matsuoka, Takashi Okabe, Yoshiaki Wakumoto, Nobumichi Tanaka, Mikio Sugimoto, Masafumi Oyama, Kiyohide Fujimoto, Shigeo Horie, Masaru Funagoshi, Ichiro Arakawa, Shinichi Noto, Kojiro Shimozuma

**Affiliations:** 1 Department of Life Sciences, Ritsumeikan University, Kusatsu, Japan; 2 Department of Urology, Hamamatsu University School of Medicine, Hamamatsu, Japan; 3 Department of Urology, Faculty of Medicine, Kagawa University, Kagawa, Japan; 4 Department of Uro-Oncology, Saitama Medical University International Medical Center, Saitama, Japan; 5 Department of Urology Juntendo University, Graduate School of Medicine, Tokyo, Japan; 6 Department of Urology, Nara Medical University, Kashihara, Japan; 7 Faculty of Pharmaceutical Science, Teikyo Heisei University, Tokyo, Japan; 8 Department of Rehabilitation, Niigata University of Health and Welfare, Niigata, Japan

**Keywords:** Health-related quality of life (HRQoL), EQ-5D-5L, Ceiling effect, FACT-P, Prostate cancer

## Abstract

**Purpose:**

The goal of the present study was to determine factors related to a ceiling effect (CE) on the EQ-5D-5L among Japanese patients with prostate cancer (PC).

**Methods:**

An existent cross-sectional observational study dataset was used. Patients were ≥ 20 years of age and diagnosed with PC. For CE determinants on the EQ-5D-5L, we excluded possible “full-health” patients flagged by the EQ-VAS (score = 100) and/or FACT-P (score = 156) instruments. We then divided them into binary variables: A CE group (EQ-5D-5L score = 1) and others (< 1). The associations between CE, sociodemographic and medical characteristics, and FACT-P subscale scores were examined using a multivariate LASSO selection followed by a binomial logistic regression analysis performed to calculate odds ratios (ORs) and 95% confidence intervals (CIs).

**Results:**

A total of 362 patients were analyzed. The LASSO selection variables, including all obtained variables, were as follows: age, palliative treatment, FACT-P physical well-being, and PC subscale score. Statistically significant variables predicting CE were palliative treatment (OR 0.23; 95% CI 0.09–0.60), physical well-being (OR 1.54; 95% CI 1.34–1.76), and PC subscale (OR 1.08; 95% CI 1.03–1.14).

**Conclusions:**

This study revealed that palliative treatment and two FACT-P physical well-being and PC subscale scores were positively related to CE on the EQ-5D-5L. To our knowledge, this is the first study to examine predictors of CE on the EQ-5D-5L. The present results may be helpful for facilitating the consideration of “bolt-on” studies from the standpoint of PC patients.

## Introduction

The EuroQol—5 Dimension (EQ-5D) is an instrument that measures generic preference-based health status and provides utility scores [1]. The EQ-5D is one of the most widely used instruments in health economic analyses [2–6]. The instrument is a utility measure comprising dimensions regarding morbidity, self-care, usual activities, pain or discomfort, and anxiety or depression. The former version, EQ-5D-3 Level (3L), is evaluated at three levels (no problems, some problems, and extreme problems) as indicated by respondents, and converted utility scores are then used by a country-specific tariff [7].

The utility scores range from 0 (dead) to 1 (perfect health), although a score less than 0 (negative) suggests a status worse than death depending on the instrument used [1]. Utility scores are multiplied by the survival year and used for a cost–utility analysis to determine quality-adjusted life years (QALYs) [1, 8].

Although one report suggests that 63.2% of studies published in the Web of Science (2004–2010) using a generic preference-based measure have included the EQ-5D [9, 10], it is well known that the EQ-5D-3L produces a ceiling effect (CE) [5, 7, 11]. This indicates a range-of-instrument constraint [12]. For this reason, the EQ-5D-5 Level (5L) was established to improve sensitivity and reduce CE. The EQ-5D-5L evaluates each dimension in a similar manner as the 3L: none, slight, moderate, severe, extreme problems, or unable. For example, code “11111” represents full health. Each number code represents a unique health status, and there are 3125 (= 5^5^) health statuses that can be evaluated, with the previous 3L only including 243 (= 3^5^) health statuses [7].

While the 5L exhibits a lower CE than the 3L, there are still reports of a CE using the 5L within general population samples [11, 12]. For instance, Konnopka and Koening [12] used the term “no-problems-problem” in a study that analyzed the association between CE and morbidity. As the EQ-5D was never intended to cover all dimensions of health due to a five-dimensional structure, there are some health problems that cannot be captured. For example, vision, hearing, and some mental health disorders [9, 13, 14] have disease-specific symptoms. This means that different types of instruments are sometimes needed [9]. Several researchers, including a working group established by the EuroQol Group (which developed the EQ-5D), are undertaking studies to examine the use of “bolt-on” dimensions [9, 13–24].

It is important to focus on the factors hidden by a CE on the EQ-5D-5L. For patients with prostate cancer (PC), Färkkilä et al. [25] used the 3L version and reported a pronounced CE, even among patients at the end-stage of the disease. In addition, our previous study used the EQ-5D-5L in a Japanese PC sample and observed a significant CE [26].

The goal of the present study was to determine factors related to a CE on the EQ-5D-5L among Japanese PC patients. To simplify this analysis, we defined the CE on the EQ-5D-5L as the sets of scores where the EQ-5D-5L scores = 1 (maximum possible score), but other health measurement instrument scores (EQ-Visual Analogue Scale (VAS) [2] and/or disease-specific Functional Assessment of Cancer Therapy-Prostate (FACT-P) [27]) did not reach the maximum. We considered this to be a possible way to determine some unhealthy factors that cannot be captured by the EQ-5D-5L. The data from a previous study [26] were used.

## Methods

### Data collection

A cross-sectional observational study was conducted that recruited PC patients from five university hospitals in Japan between February and December 2017. Although the method of data collection to obtain health utility and health-related quality of life data in this sample is reported elsewhere [26], it is described again in this article, below. Patients were ≥ 20 years of age and diagnosed with PC at each hospital. These data were collected in agreement with the principles of the Declaration of Helsinki and approved by the Ethics Committee of Ritsumeikan University (BKC-2016-042) and each participating hospital. One hundred patients were registered at each hospital (n = 500 in total). Informed consent was obtained from 493 of these patients, and 453 completed questionnaires were returned by participants. Of these, we could not obtain 69 scores due to missing answers on the EQ-5D-5L and/or FACT-P scoring questionnaire(s). Furthermore, 4 patients could not be stratified into a cancer progression group due to missing information in their physician report. In addition, for this present research, we excluded 18 patients who had a maximum score on the EQ- VAS (score = 100) [2] and/or FACT-P (score = 156). This was done in order to exclude possible “full-health” patients flagged by these two instruments. This means that only the data from patients who did not receive the maximum possible scores on the EQ-VAS and/or FACT-P but did receive the maximum on the EQ-5D-5L were examined. Finally, data from 362 patients were used in this study.

### Questionnaires

Patients responded to self-administered questionnaires on sociodemographic, EQ-5D-5L, EQ-VAS, and FACT-P instruments [26]. Self-rated answers by each patient for the EQ-5D-5L were converted to utility scores by the Japanese tariff [5]. For FACT-P, Japanese FACT-P (version 4) questionnaires were used and scored in accordance with the instructions provided by the Functional Assessment of Chronic Illness Therapy (FACIT) [28]. The sociodemographic questions comprised birth date, family members, education level, job, income, and other diseases. These questionnaires were mostly offered in multiple-choice formats. Medical information was provided by their physicians in charge after informed consent was given, and the filled-out questionnaires were sent by mail and returned by patients to the data center [26]. The provided information included the presence or absence of cancer progression, prostate-specific antigen (PSA) concentration [29–31], number of days from last treatment, the presence or absence of other diseases, and Eastern Cooperative Oncology Group (ECOG) performance status (PS) [32–34], and common terminology criteria for adverse events (CTCAE) version 4.0 [35] were collected. In addition, we added information regarding received treatments (i.e., treatments previously undertaken and ongoing treatments) in order to examine therapies related to a CE for this analysis. This medical information was also provided by physicians in charge. A report form was asked about current and past treatments, as well as the number of days since the most recent current treatment. For received treatments, physicians in charge were selected from the following areas: surgery, chemotherapy, hormonal therapy, external-beam radiation therapy, brachytherapy, active surveillance, watchful waiting, and palliative treatment. All obtained variables are listed in Table 1.

**Table 1:** Patient sociodemographic and medical characteristics and FACT-P subscale scores based on the EQ-5D-5L, *n* (% or mean ± SD)

Variable	EQ-5D-5L		Total *n* = 362
	Score < 1 *n* = 187	Score = 1 *n* = 175	
Sociodemographic characteristics			
Age (mean ± SD years)	74.5 ± 7.9	71.9 ± 7.3	73.2 ± 7.8
Highest education^a^			
Junior high school or less	44 (23.9)	34 (19.9)	78 (22.0)
High school	66 (35.9)	59 (34.5)	125 (35.2)
College or more	74 (40.2)	78 (45.6)	152 (42.8)
Income^a, b, c^			
≤ ¥3,000,000/year	64 (37.0)	51 (30.2)	115 (33.6)
> ¥3,000,000/year to ≤ ¥5,000,000/year	65 (37.6)	74 (43.8)	139 (40.6)
> ¥5,000,000/year	44 (25.4)	44 (26.0)	88 (25.7)
Job changes^d^	58 (35.4)	59 (38.1)	117 (36.7)
Living with^a^			
Wife	102 (54.8)	94 (54.0)	196 (54.4)
Wife and other family member(s)	58 (31.2)	62 (35.6)	120 (33.3)
Alone	20 (10.8)	9 (5.2)	29 (8.1)
Other	6 (3.2)	9 (5.2)	15 (4.2)
Medical characteristics			
PC progression status			
Localized	133 (71.1)	133 (76.0)	266 (73.5)
Localized progression	20 (10.7)	18 (10.3)	38 (10.5)
Distant metastatic	13 (7.0)	8 (4.6)	21 (5.8)
Distant metastatic castration-resistant	21 (11.2)	16 (9.1)	37 (10.2)
PSA concentration (mean ± SD ng/mL)	11.5 ± 54.2	7.1 ± 49.1	9.4 ± 51.8
Suffering other disease(s)^e^	180 (96.3)	172 (98.3)	352 (97.2)
ECOG performance status^a^			
0	146 (81.6)	158 (92.4)	304 (86.9)
1	28 (15.6)	11 (6.4)	39 (11.1)
≥ 2	5 (2.8)	2 (1.2)	7 (2.0)
Maximal CTCAE grade			
0	92 (49.2)	91 (52.0)	183 (50.6)
1	52 (27.8)	40 (22.9)	92 (25.4)
≥ 2	43 (23.0)	44 (25.1)	87 (24.0)
Days from last treatment (mean ± SD days)	3.0 ± 11.1	8.2 ± 23.5	5.5 ± 18.4
Received treatment^f^			
Hormonal therapy	97 (51.9)	87 (49.7)	184 (50.8)
Surgery	30 (16.0)	23 (13.1)	53 (14.6)
Active surveillance	21 (11.2)	24 (13.7)	45 (12.4)
External-beam radiation therapy	23 (12.3)	17 (9.7)	40 (11.1)
Brachytherapy	19 (10.2)	20 (11.4)	39 (10.8)
Palliative treatment	18 (9.6)	10 (5.7)	28 (7.7)
Chemotherapy	15 (8.0)	11 (6.3)	26 (7.2)
Watchful waiting	2 (1.1)	3 (1.7)	5 (1.4)
FACT-P subscale scores (mean ± SD)			
Physical well-being	22.5 ± 4.3	26.7 ± 1.7	24.5 ± 3.9
Social well-being	14.5 ± 6.8	14.8 ± 7.7	14.6 ± 7.2
Emotional well-being	17.8 ± 4.1	20.3 ± 2.8	19.0 ± 3.7
Functional well-being	17.1 ± 6.3	20.5 ± 7.2	18.7 ± 7.0
PC subscale	30.3 ± 7.0	36.4 ± 5.3	33.2 ± 6.9
Registered hospital^b^			
Hospital A	37 (19.8)	37 (21.1)	74 (20.4)
Hospital B	32 (17.1)	40 (22.9)	72 (19.9)
Hospital C	37 (19.8)	27 (15.4)	64 (17.7)
Hospital D	36 (19.3)	42 (24.0)	78 (21.5)
Hospital E	45 (24.1)	29 (16.6)	74 (20.4)

*SD* standard deviation, *PC* prostate cancer, *PSA* prostate-specific antigen [29–31], *ECOG* Eastern Cooperative Oncology Group [32–34], *CTCAE* common terminology criteria for adverse events [35]

^a^Does not match with the total number due to missing information

^b^Does not total 100% due to rounding

^c^Exchange rate was 1 USD = 113 JPY in December 2017

^d^Patients who changed their job following their diagnosis

^e^Includes hypertension, diabetes, hyperlipidemia, other cancer, and prostatic hypertrophy

^f^Allows multiple selection and includes both current and past treatments

### Statistical analyses

Data were divided into two groups according to the EQ-5D-5L score, those with a score = 1 (CE group) and others. Because the data used already excluded maximum scores on the EQ-VAS and/or FACT-P (possible “full-health”) patients, we could thus categorize the remaining participants into two groups: one with EQ-5D-5L scores < 1 and the other a CE group that has some unhealthy factors that cannot be captured by the EQ-5D-5L but can be captured by the EQ-VAS and/or FACT-P. Spearman’s correlation coefficients were calculated between the EQ-5D-5L and FACT-P scores to check for any associations between instruments.

Possible predictors for a CE among sociodemographic and medical factors were explored using Least　Absolute Shrinkage and Selection Operator (LASSO) methods based on minimal Akaike’s Information Criterion (AIC), which set these factors as independent variables. Next, binomial logistic regression analyses with the selected variables were performed to obtain each odds ratio (OR) for factors adopted in the models. Model 1 was used for sociodemographic characteristics, Model 2 for medical status, Model 3 for FACT-P subscale scores, Model 4 for sociodemographic characteristics and medical status, and Model 5 for all factors. Registered hospitals were set as dummy variables in all LASSO selections to choose and adjust for potential differences such as a selection bias among them.

The 95% confidence interval (CI) was used to determine statistical significance of the OR. JMP Pro 14.1.0 (SAS Institute Inc. Cary, NC, USA) was used for all analyses.

## Results

**Fig. 1:**
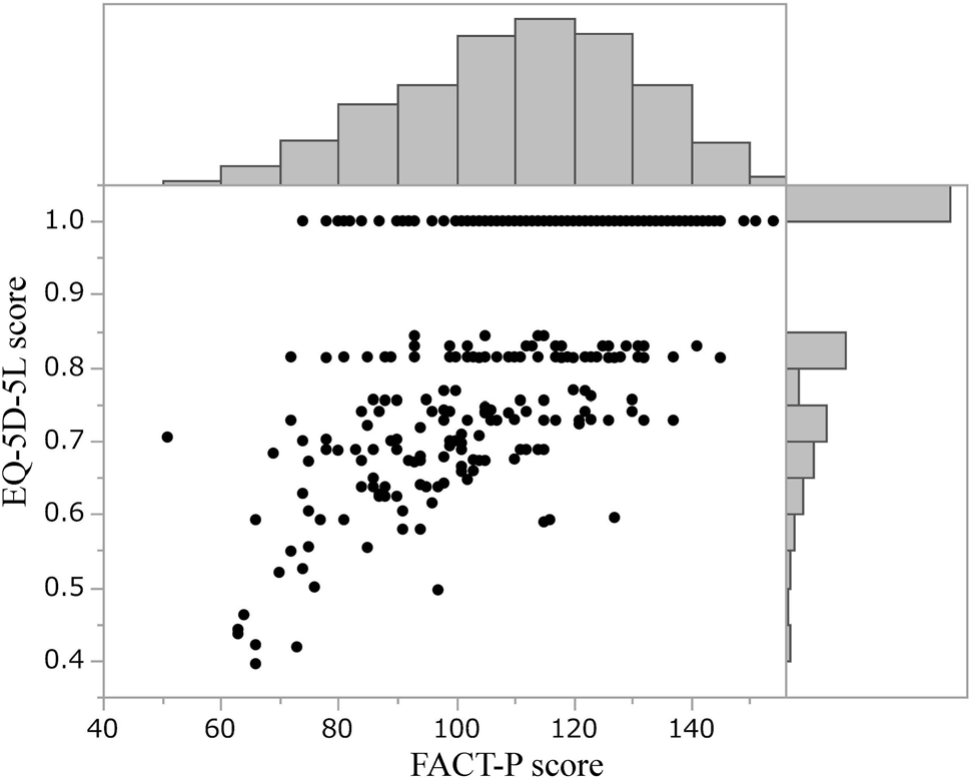
Scatterplot for EQ-5D-5L versus FACT-P scores. Marginal histograms of these scores are placed. 48.3% of patients had an EQ-5D-5L score = 1. The Spearman’s rank correlation coefficient between the scores of these two instruments was 0.52 (*n* = 362)

Of the 362 patients, 48.3% (*n* = 175) had an EQ-5D-5L score = 1. The plots for patients’ EQ-5D-5L and FACT-P scores are shown in Fig. 1. The Spearman’s rank correlation coefficient between the two instruments’ scores was 0.52. The sociodemographic and medical characteristics and FACT-P subscale scores according to CE are shown in Table 1.

The LASSO selections included sociodemographic and medical characteristics and FACT-P subscale scores for Model 1, Model 2, and Model 3. Selected variables used for the multivariate binomial logistic regression analyses predicting CE are shown in Table 2. In addition, results from Model 4 for sociodemographic and medical factors, and Model 5 for all factors (sociodemographic, medical, and FACT-P subscale scores), are shown.

**Table 2:** Multivariate-adjusted ORs and 95% CIs for factors associated with EQ-5D-5L CE

Variable	Model 1		Model 2		Model 3 363.2		Model 4		Model 5	
	AIC = 492.7		AIC = 466.5		AIC = 363.2		AIC = 458.9		AIC = 352.7	
	OR	(95% CI)	OR	(95% CI)	OR	(95% CI)	OR	(95% CI)	OR	(95% CI)
Sociodemographic characteristics										
Age	**0.96**	**(0.93, 0.98)**	**–**		**–**		**0.95**	**(0.92, 0.98)**	0.97	(0.94, 1.00)
Highest education										
Junior high school or less	NA		**–**		**–**		NA		NA	
High school	NA		**–**		**–**		NA		NA	
College or more	NA		**–**		**–**		NA		NA	
Income										
≤ ¥3,000,000/year	NA		**–**		**–**		NA		NA	
> ¥3,000,000/year – ≤ ¥5,000,000/year	NA		**–**		**–**		NA		NA	
> ¥5,000,000/year	NA		**–**		**–**		NA		NA	
Job changes	NA				**–**		NA		NA	
Living with										
Wife	Ref.		**–**		**–**		Ref.		NA	
Wife and other family member(s)	1.02	(0.64, 1.63)	**–**		**–**		1.09	(0.65, 1.82)	NA	
Alone	0.48	(0.21, 1.14)	**–**		**–**		**0.33**	**(0.12, 0.88)**	NA	
Other	1.55	(0.52, 4.59)	**–**		**–**		1.84	(0.56, 6.01)	NA	
Medical characteristics										
PC progression status										
Localized	**–**		NA		**–**		NA		NA	
Localized progression	**–**		NA		**–**		NA		NA	
Distant metastatic	**–**		NA		**–**		NA		NA	
Distant metastatic castration-resistant	**–**		NA		**–**		NA		NA	
PSA concentration	**–**		NA		**–**		1.00	(0.99, 1.00)	NA	
Suffering other disease(s)	**–**		NA		**–**		NA		NA	
ECOG performance status										
0	**–**		Ref.		**–**		Ref.		NA	
1	**–**		**0.17**	**(0.07, 0.40)**	**–**		**0.20**	**(0.08, 0.52)**	NA	
≥ 2	**–**		0.21	(0.04, 1.22)	**–**		0.21	(0.02, 2.14)	NA	
Maximal CTCAE grade										
0	**–**		NA		**–**		Ref.		NA	
1	**–**		NA		**–**		0.79	(0.40, 1.54)	NA	
≥ 2	**–**		NA		**–**		1.06	(0.50, 2.23)	NA	
Days from last treatment	**–**		**1.02**	**(1.00, 1.04)**	**–**		**1.02**	**(1.00, 1.04)**	NA	
Received treatment										
Hormonal therapy	**–**		NA		**–**		NA		NA	
Surgery	**–**		0.53	(0.28, 1.04)	**–**		**0.39**	**(0.19, 0.78)**	NA	
Active surveillance	**–**		NA		**–**		NA		NA	
External-beam radiation therapy	**–**		**0.40**	**(0.18, 0.86)**	**–**		**0.40**	**(0.18, 0.90)**	NA	
Brachytherapy	**–**		NA		**–**		NA		NA	
Palliative treatment	**–**		0.53	(0.23, 1.23)	**–**		**0.35**	**(0.14, 0.88)**	**0.23**	**(0.09, 0.60)**
Chemotherapy	**–**		NA		**–**		NA		NA	
Watchful waiting	**–**		NA		**–**		NA		NA	
FACT-P subscale scores										
Physical well-being	**–**		**–**		**1.54**	**(1.35, 1.76)**	**–**		**1.54**	**(1.34, 1.76)**
Social well-being	**–**		**–**		NA		**–**		NA	
Emotional well-being	**–**		**–**		NA		**–**		NA	
Functional well-being	**–**		**–**		NA		**–**		NA	
PC subscale	**–**		**–**		**1.07**	**(1.02, 1.13)**	**–**		**1.08**	**(1.03, 1.14)**
Registered hospital^a^										
Hospital A	NA		1.70	(0.91, 3.17)	NA		**2.60**	**(1.17, 5.74)**	NA	
Hospital B	NA		**4.11**	**(1.91, 8.85)**	NA		**5.48**	**(2.18, 13.8)**	NA	
Hospital C	NA		NA		NA		1.75	(0.82, 3.74)	NA	
Hospital D	NA		1.61	(0.87, 2.96)	NA		2.20	(0.99, 1.01)	NA	

Bold characters represent significance of the OR determined by 95% CI

*PSA* prostate-specific antigen [29–31], *OR* Odds ratio, *CI* Confidence interval, *CE* ceiling effect, *AIC* Akaike’s Information Criterion, *PC* prostate cancer, *ECOG* Eastern Cooperative Oncology Group [32–34], *CTCAE* common terminology criteria for adverse events [35], *NA* Factors not assessed by logistic regression models due to the results of LASSO selections—factors not input into a LASSO selection

^a^Registered hospitals were set as dummy variables in all LASSO selections (Hospital A to D were set in four binomial (0 or 1) variables; An instance of all variables being 0 is represented by Hospital E)

For Models 1, 2, and 3, the adjusted ORs revealed the following statistically significant predictors: age (OR 0.96; 95% CI 0.93–0.98 in Model 1), ECOG PS = 1 (OR 0.17; 95% CI 0.07–0.40 in Model 2), days from last treatment (OR 1.02; 95% CI 1.00–1.04 in Model 2), external-beam radiation therapy (OR 0.40; 95% CI 0.18–0.86 in Model 2), FACT-P physical well-being subscale score (OR 1.54; 95% CI 1.35–1.76 in Model 3), and PC subscale score (OR 1.07; 95% CI 1.02–1.13 in Model 3). In Model 4, all variables selected in Models 1 and 2 were also selected. The statistically significant ORs (95% CIs) in Model 4 for age, living alone, ECOG PS = 1, days from last treatment, surgery, external-beam radiation therapy, and palliative treatment were 0.95 (0.92–0.98), 0.93 (0.12–0.88), 0.20 (0.08–0.52), 1.02 (1.00–1.04), 0.39 (0.19–0.78), 0.40 (0.18–0.90), and 0.35 (0.14–0.88), respectively. In addition, one and two dummy variables for registered hospitals showed statistically significant ORs in Models 2 and 3, respectively.

Model 5 had the lowest AIC among all five models. Here, palliative treatment (OR 0.23; 95% CI 0.09–0.60), FACT-P physical well-being subscale score (OR 1.54; 95% CI 1.34–1.76), and PC subscale score (OR 1.08; 95% CI 1.03–1.14) were significant.

## Discussion

The present study was performed in order to determine factors associated with an EQ-5D-5L CE among Japanese patients with PC [26]. We excluded patients with a maximum score on the EQ-VAS and/or FACT-P in order to remove possible “full-health” patients. This exclusion ensured that the patient sample was experiencing health-related problems that may not be detected by the EQ-5D-5L but could be revealed via alternative instruments.

Among the sociodemographic and medical characteristics examined in Models 1, 2, and 4 (Table 2), age, living alone, ECOG PS = 1, and received treatments (surgery, external-beam radiation therapy, and palliative treatment) were negatively related to a lower CE. The aging process is associated with deteriorated health status and diminished HRQoL. For instance, even among a random sample of Japanese adults, QoL scores are lower for individuals over the age of 60 [36]. In addition, Konnopka and Koeing [12] observed significant associations between age and the EQ-5D-5L dimensions of “morbidity” and “self-care.” In terms of living alone, one study analyzed the associations between living arrangement and HRQoL, revealing that HRQoL among older adults living alone or with adult children was worse than those living with a spouse in an urban area [37]. In addition to living alone, quality of care and support is also associated with HRQoL [26]. Regarding ECOG PS scores [33], a score of 0 or 4 is given when a patient is “fully active, able to carry on all pre-disease activities without restriction” or “completely disabled; cannot carry out any self-care; totally confined to a bed or chair,” respectively. These items are very similar to the EQ-5D domains of morbidity and usual activities. Thus, a high score on the ECOG PS correlates with the EQ-5D. Among the received treatments, three were negatively associated with a CE. Although further analyses are needed, namely at the EQ-5D domain level, these treatments are likely to have negative consequences for patients when compared to other treatments (e.g., active surveillance and/or brachytherapy).

The days from last treatment variable was positively significant in Models 2 and 4. A recent longitudinal study on HRQoL among locally advanced or advanced PC patients was reported by Zajdlewicz et al. [38]. Though their study did not use the EQ-5D and FACT-P, HRQoL fluctuated from diagnosis to a 5-year follow-up assessment. The significance observed in Models 2 and 4 in the present study disappeared when FACT-P subscale scores were added. Thus, it seems that this association may be related to specific FACT-P factors.

Model 3 included FACT-P subscale scores, specifically physical well-being and PC subscale scores. Two prior studies analyzed FACT-P to EQ-5D-3L mapping in metastatic PC. One was a study using data from AFFIRM (A Study Evaluating the Efficacy and Safety of the Investigational Drug MDV3100) [39]. Physical well-being and the PC subscale had the highest predictive value among a healthy participant sample (FACT-P score ≥ 76). The other study was conducted in six European countries. The physical and functional well-being subscales had the highest predictive value for the EQ-5D based on generalized linear models [40]. The discrepancies between these two studies could be due to the analyses and settings employed. Although our results showed significance differences of physical well-being and the PC subscale, just as the AFFIRM study showed, further work is needed to examine similarities and differences between FACT-P and EQ-5D-5L associations.

When we added FACT-P subscales into the sociodemographic and medical factors models, results changed considerably (Model 4 to Model 5 in Table 2). The variables of living statuses, PSA concentration, ECOG PS, maximal CECAE grades, the days from last treatment, surgery, external-beam radiation therapy and dummy variables for registered hospitals were not selected, and age was no longer significant. However, the physical well-being and PC subscale score significance was maintained from Model 3 to Model 5. It is possible that the continuity in significance values could be due to the highly sensitive nature of the FACT-P subscales.

Statistical significance for palliative treatment was observed in Models 4 and 5. Palliative treatment includes care for pain stemming from bone metastasis, spinal paralysis, hematuria, lower urinary tract obstruction, and renal failure [30]. These symptoms work to negatively impact the ceiling effect of EQ-5D-5L. Further studies are needed to clarify which circumstances, situations, and/or statuses caused by the palliative treatment can be explained by factors other than the two FACT-P subscale scores. This viewpoint may also be helpful for facilitating “bolt-on” studies concerning PC patients.

Although Konnopka and Koenig [12] focused on the association between CE on the EQ 5D-5L and morbidity in a general population, to our knowledge, ours is the first study to analyze the association between CE on the EQ-5D-5L and predictor variables among PC patients. Nevertheless, we would like to raise four limitations of this study. First, due to the small sample size, we could not perform more detailed analyses, such as those performed by Konnopka and Koenig (i.e., analyzing the EQ-5D-5L at the dimension level). To better clarify and discuss our afore-mentioned gaps (i.e., which factors contributed to the loss of significance for the living alone, surgery, external-beam radiation therapy variables, etc.), analyses at the EQ-5D-5L domain level and with better statistical power are needed. Second, as we have described elsewhere [26], the present study relied on self-administered questionnaires. We cannot completely rule-out the possibility of selection bias. If patients were unable to complete the questionnaires, they were excluded for practical and ethical purposes. Third, we implemented five LASSO selection and followed logistic regression models. While cautions must be taken for multicollinearity and confounding in multivariate analysis, it has been reported that LASSO selection performs relatively better concerning multicollinearity and robustness [41–44]. Model 5 showed the lowest AIC among our models, and almost all inputted variables were excluded, which indicates that the LASSO selections in our analysis work well. Lastly, statistically significant ORs of dummy variables for registered hospitals in a few of the models may have occurred due to the different tendency for disease progression of patients registered in each hospital [26]. We also cannot exclude regional differences between the hospitals and/or judgments by physicians in charge. Therefore, there is a possibility that our results do not sufficiently generalize to the entire population of PC patients.

## Conclusions

The present study revealed that palliative treatment and two FACT-P subscale scores (physical well-being and PC subscale) were positively related with a CE on the EQ-5D-5L. These results indicate the importance of instruments not only for generic preference-based utility for the EQ-5D but also the necessity of disease-specific HRQoL instruments (i.e., the FACT-P) to assess health status. In addition, the present results may also be helpful for facilitating the consideration of “bolt-on” studies from the standpoint of PC patients.
